# Failure Mode Analysis of an Exeter Stem Fracture Initiated at the Introducer Hole: Time for a Design Change?

**DOI:** 10.1016/j.artd.2021.07.016

**Published:** 2021-10-04

**Authors:** Ralph M. Jeuken, Duncan P. Fransz, Marc G.D. Geers, Marc P.F.H.L. van Maris, René H.M. ten Broeke

**Affiliations:** a Department of Orthopaedics, Maastricht University Medical Center, Maastricht, Limburg, the Netherlands; b Department of Orthopaedics, Zuyderland Medical Center, Heerlen, Limburg, the Netherlands; c Department of Mechanical Engineering, Eindhoven University of Technology, Eindhoven, Noord-Brabant, the Netherlands

**Keywords:** Case report, Hip, Prosthesis, Failure, Metallurgy

## Abstract

The fracturing of a hip prosthesis stem at its neck, in the absence of a trauma, is an extremely rare but serious adverse event. The patient in our case was young, active, and tall, thereby putting high mechanical loads on the prosthesis. Radiographs of the initial procedure and blood and synovium analysis showed no abnormalities. Analysis of the stem revealed niobium-rich precipitates, that is, alloy artifacts, at the introducer stud hole. The mechanically vulnerable location of the introducer stud hole, combined with alloy artifacts at that location and high mechanical stress, ultimately led to failure of the prosthesis. As younger and heavier patients will demand hip arthroplasty in the future, simple stem design adaptations should be considered to prevent stem fractures at the introducer stud hole.

## Introduction

Total hip arthroplasty (THA) is one of the most commonly performed procedures in orthopedic surgery and is one of the most successful major surgeries of modern medicine [1]. The Exeter femoral stem (Stryker, Newbury, UK) was first introduced in 1969-1970, and while its design has gone through 4 design changes, it remains one of the most successful and most used cemented stems [2]. The current Exeter V40 stem was introduced in 2001 and has a smaller taper with a reduction in both taper tip diameter and taper length compared to its predecessor [3]. Failure percentages are very low [4,5]; however, they do matter given the high number of implantations performed annually and its projected rise [6]. In case of implant failure, the 3 major causes typically are dislocation, mechanical loosening, and periprosthetic joint infection [4].

Although very unlikely, a femoral stem can fracture *in vivo* because of failure of the metal alloy. A recent study of 80 retrieved fractured Exeter prostheses in the period 1991-2008 showed that this type of fracture is very rare, with a minimum reported failure rate of 1 in 10.000 stems (0.01%) [2]. Other studies reported an estimated minimum rate of mechanical failure of 0.2% [7] or estimated the overall risk of a stem fracture at 0.262% [8]. The 2 main fracture sites identified are the body (54%) or the neck (46%) of the stem. With regard to the neck fractures, a subcapital (77%), or a basal fracture initiating at the introducer hole (23%) has been recorded [2].

Whenever an implant fails, it is common practice that the manufacturer retrieves the implant for further analyses [2]. As such a failure is multifactorial (ie, implant, patient, and surgical factors), information from all 3 areas should be identified and scrutinized [2].

In this case report, we set out to synthesize all the available and obtainable information on a recent patient case that suffered from an Exeter stem fracture that initiated at the introducer hole. The unique “perfect storm” that, in this case, ultimately led to failure of the Exeter femoral prosthesis provides important implications for future stem designs and surgical considerations.

## Case history

### Patient

**Figure 1:**
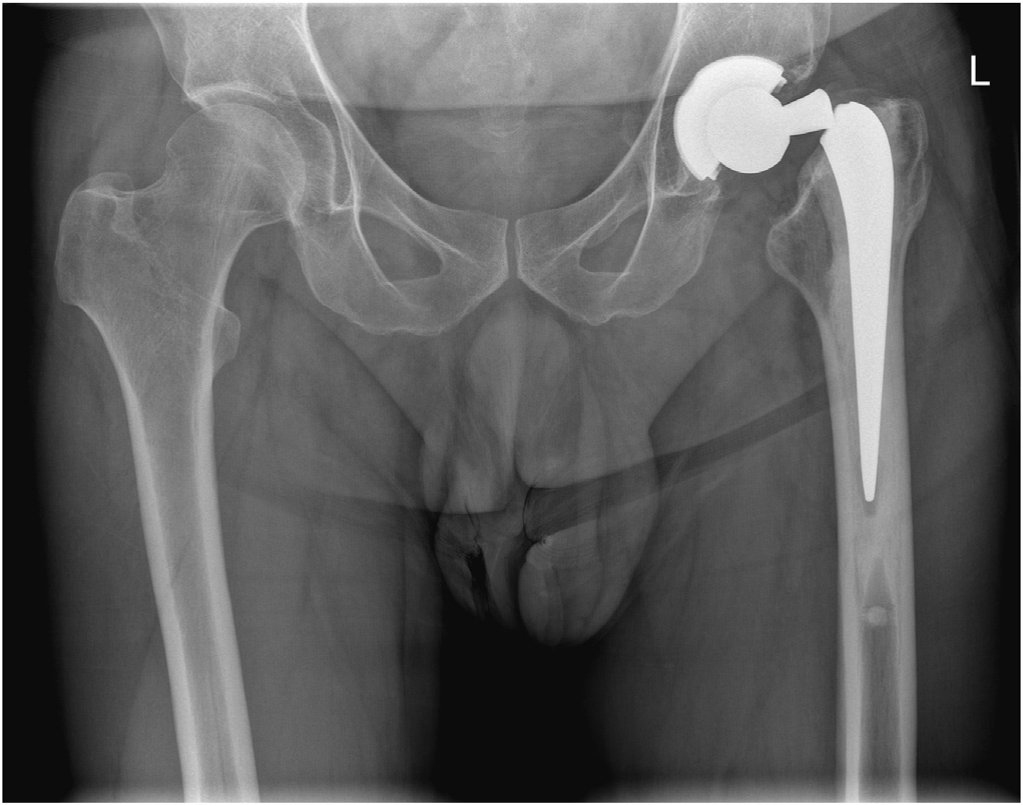
Anteroposterior pelvic plain radiograph showing the neck fracture of the cemented Exeter stem.

A healthy and active 44-year-old male patient (height 1.90 m; weight 95.0 kg; body mass index 26.3) presented at our emergency department with pain in the left groin or hip. A sudden “crack” had occurred during a low-speed pivoting movement while working as a kitchen chef and left him unable to bear weight. Exactly 6 years earlier, the patient had been treated for a 4-week-old fracture of the left hip with hybrid THA (Tritanium solid back size-52 cup; Exeter 44-3 stem; 32 + 0 LFIT head); the stem was cemented at that time because of the superior survival of cemented stems after femoral neck fractures [9]. The obtained radiographs showed a fracture of the Exeter stem at the neck (Fig. 1), and after discussion and consent, the patient was scheduled for revision arthroplasty. After the revision surgery, the patient was satisfied with no pain or functional limitations up to his latest follow-up at 2 years postoperatively. Informed written consent was obtained from the patient to publish his personal and clinical details. Approval for the study by the local institutional review board was not required because it was a case report.

### Perioperative findings

**Figure 2:**
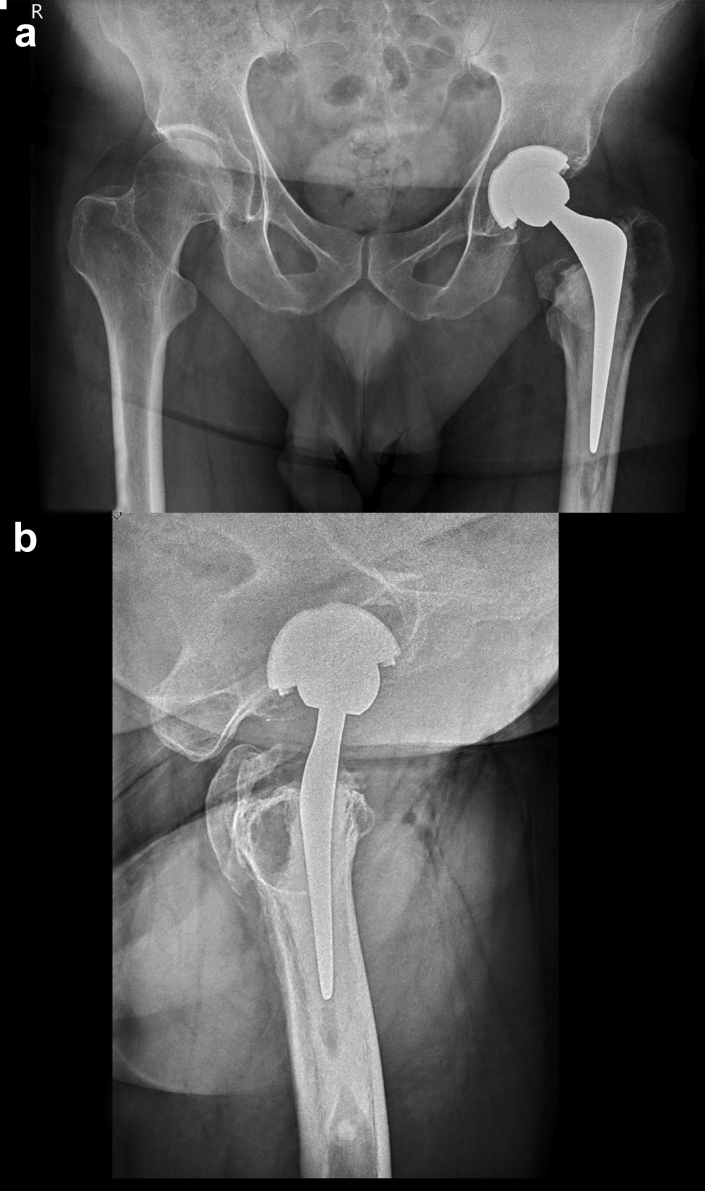
Plain anteroposterior (a) pelvic and lateral (b) hip radiographs after the revision surgery. Note the smaller implant compared to figure 3.

Intraoperatively, the *in situ* stem fragment appeared well-fixed without signs of loosening or infection. The proximal neck fragment and taper were well-engaged with the femoral head. The acetabular component had an intact rim with no signs of neck impingement, no local display of metallosis, and no evidence of eccentric wear of the polyethylene liner. The stem was removed without damaging the intact cement mantle. In brief, a hole was drilled (see the asterisk in Fig. 5) in the protruding shoulder of the Exeter stem which allowed the engagement of a clamp and linear explantation using a sliding hammer on the clamp. The femoral component was revised (cement-in-cement technique) using a smaller stem (Exeter 125-mm 44 offset, Biolox 32 + 0). No changes to the acetabular component were made (Fig. 2).

During the procedure, we collected samples of joint fluid and capsule tissue. Joint fluid was sent for biochemical analysis together with blood samples. Capsule tissue was sent for histopathological analysis.

After explantation, the stem was thoroughly cleaned with tap water and absolute ethanol to facilitate further analysis.

### Outcome and follow-up

The patient was discharged after 3 days, free of pain, fully able to bear weight, and with a dry wound. At 12 weeks, he reported difficulty “trusting his hip joint,” and at 6 months, he experienced no functional impairments. At 2 years postoperatively, the patient was functioning well with no complaints of the hip.

### Postoperative analysis

#### Radiography

**Figure 3:**
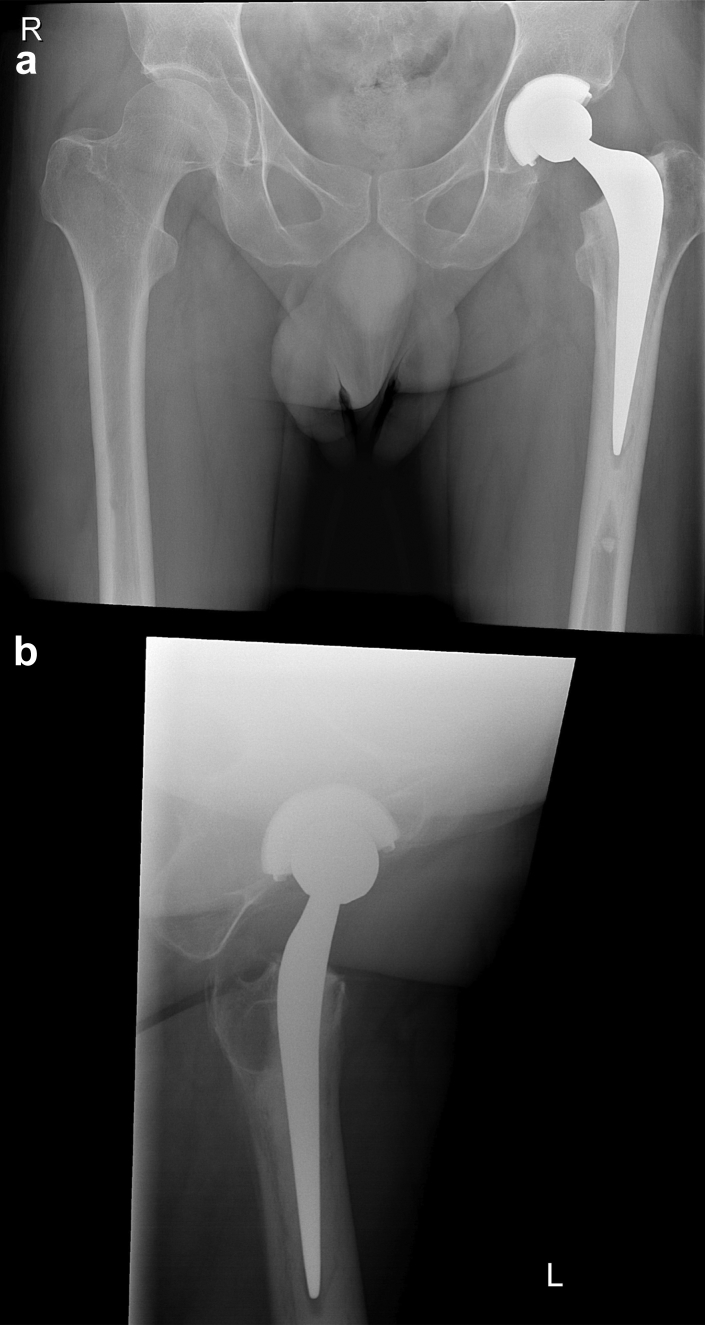
Plain anteroposterior (a) pelvic and lateral (b) hip radiographs of the index surgery showing Barack type A cementing “white out” mantle with a central position of the prosthesis in both planes.

The direct postoperative radiographs of the index operation (ie, 5.5 years before the stem fracture) are shown in Figure 3. Radiological assessment showed neutral stem position, symmetric depth (no leg length discrepancy), and adequate integrity of medial support (medial cement mantle >2 mm and sufficient medial calcar bone stock). Two years after the revision surgery, the follow-up radiograph showed a Barrack type A cementing “white out” mantle with a central position of the prosthesis in both planes.

### Biochemistry

An overview of the blood metal ion concentrations over time is shown in Table 1. All serum levels decreased over time and remained below all available reference values.

**Table 1:** Blood metal ion concentrations sampled directly after failure and at 6 and 24 months after revision surgery.

Element	At the time of revision surgery	At 6 months postoperatively	At 24 months postoperatively	Reference value^a^	Reference value^b^	Reference value^c^
Cobalt (Co)	15.1	7.5	4.5	119	30	27
Chromium (Cr)	15.9	15.9	6.0	134.5	45	29
Nickel (Ni)	39.6	32.6	17.9	—	—	—

All values are reported in nmol/L.

a Reference value of the UK Medicines and Healthcare products Regulatory Agency below which significant local soft-tissue reaction and tissue damage is suggested unlikely (10).

b Typical blood levels of well-functioning metal-on-metal (MoM) hip prosthesis according to Sampson and Hart (11). The MoM hip prosthesis typically leads to more wear particles than the cemented Exeter prosthesis with polyethylene liner.

c Highest values found in 20 analyzed patients with asymptomatic Exeter prosthesis 1 year after surgery. There are currently no available threshold reference values for systemic health effects (12). No reference values were available for nickel.

### Histopathology

**Figure 4:**
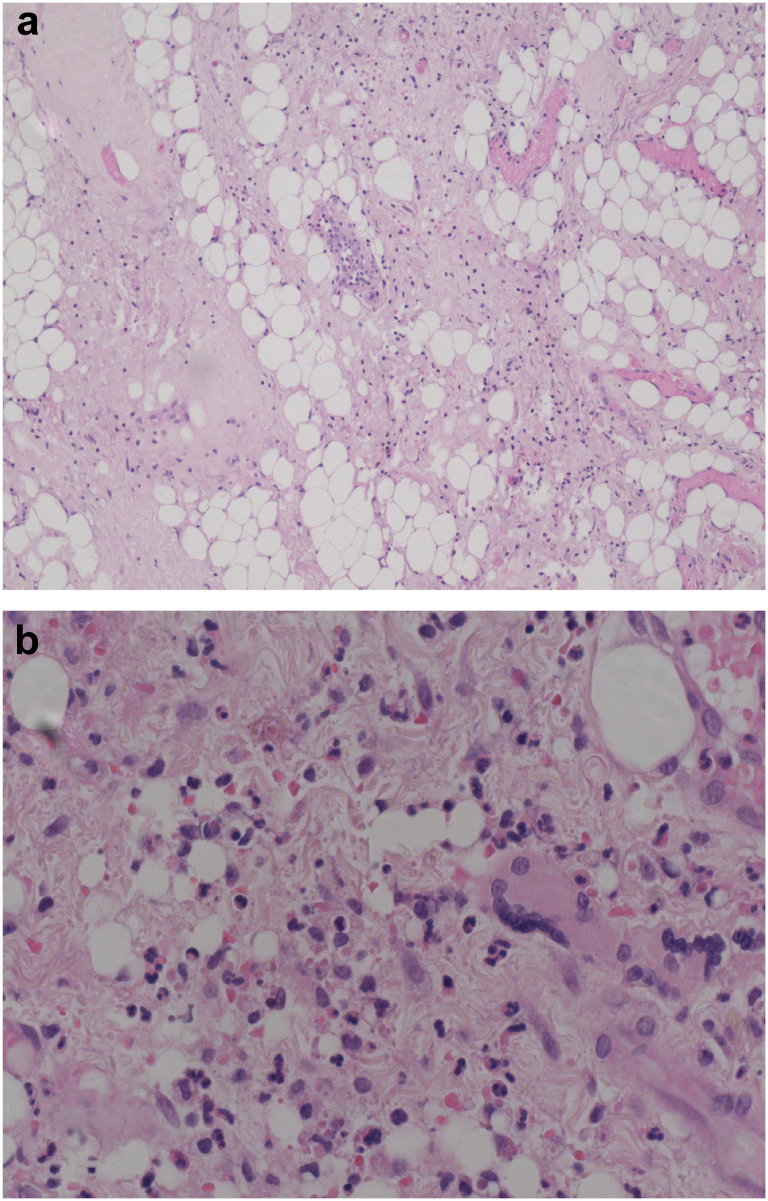
Standard hematoxylin and eosin-stained joint capsule slides at 10x (a) and 40x (b) magnification showing degenerative fibrinoid and congestive changes of the capsule and the presence of a moderate infiltrate of lymphocytes, neutrophils, and some eosinophilic granulocytes in between some nuclear debris.

Histopathology of the obtained capsule tissue (Fig. 4) shows degenerative fibrinoid and congestive changes of the capsule and the presence of a moderate infiltrate of lymphocytes, neutrophils, and some eosinophilic granulocytes in between some nuclear debris. A few giant cells are present; however, there are no metal particles.

### Metallography

**Figure 5:**
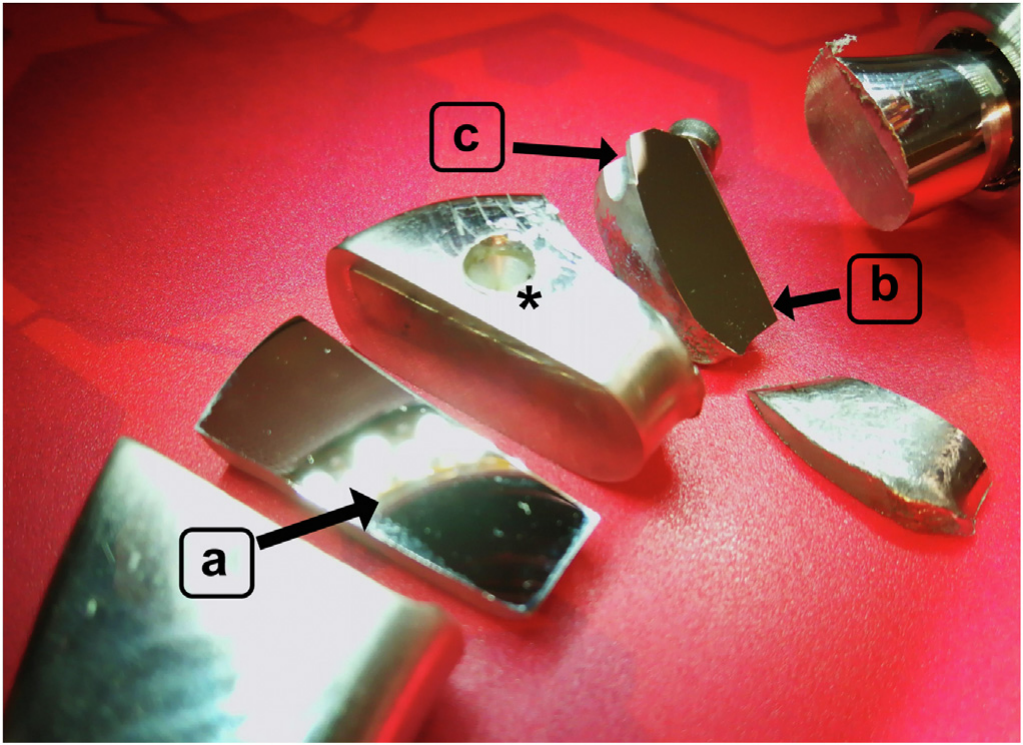
The explanted implant processed for further analysis. Samples were taken from 3 different locations: (a), at the stem away from the fractured surface; (b), surface perpendicular to the initiated crack near the stud hole; (c), surface perpendicular to the stud hole. ∗ Drilled hole to engage a clamp for explantation.

Cross-sections of the implant were processed at the Eindhoven University of Technology using successive grinding and polishing of the surfaces to obtain a high-quality surface finish as required for orientation imaging microscopy. Samples were taken from 3 different locations (Fig. 5):

A: at the stem away from the fractured surface;
B: surface perpendicular to the initiated crack near the stud hole;
C: surface perpendicular to the stud hole.

**Figure 6:**
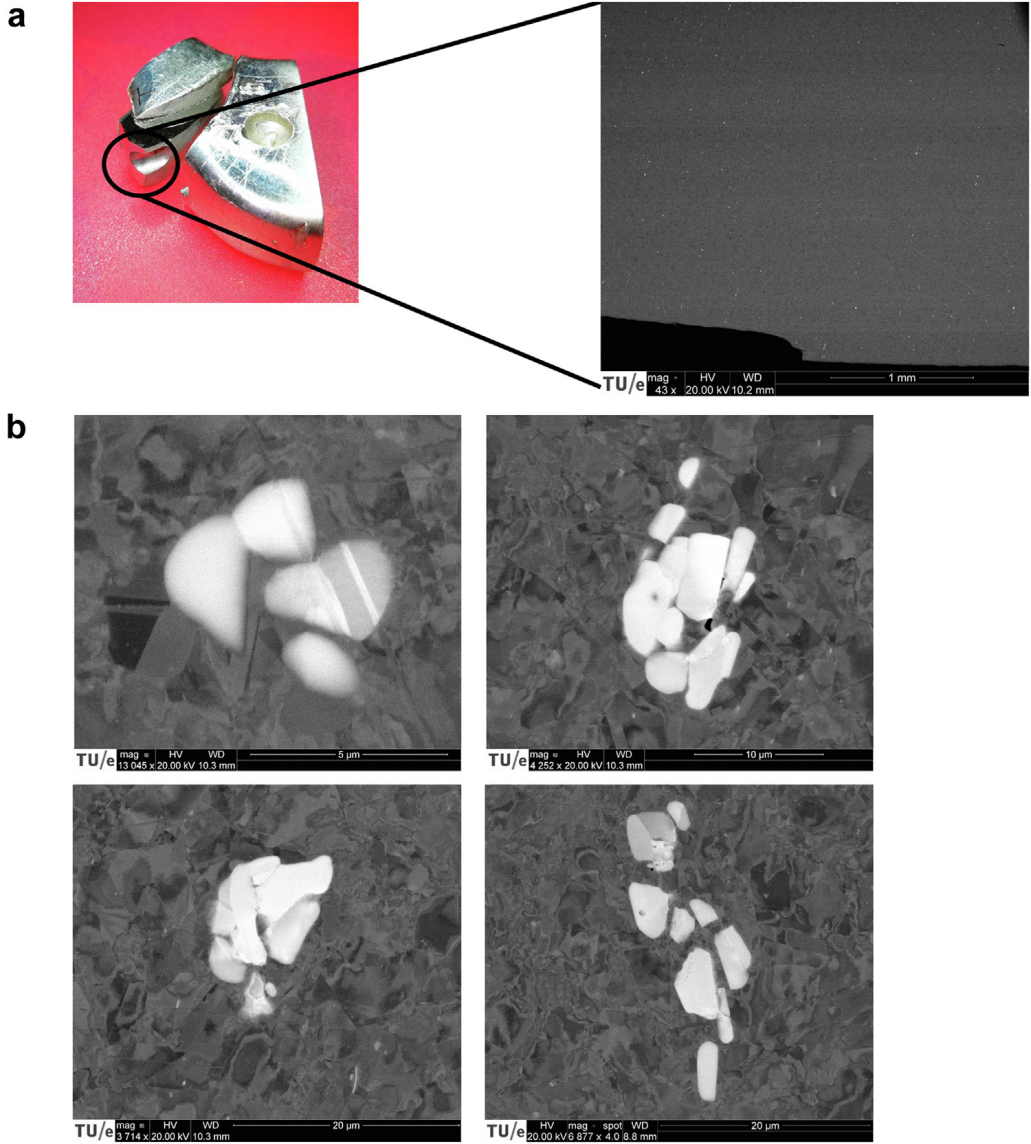
Scanning electron microscopy analysis of cross-section B (the surface perpendicular to the initiated crack near the stud hole) showing niobium (Nb)-rich phases (a) and on higher magnification (b).

**Figure 7:**
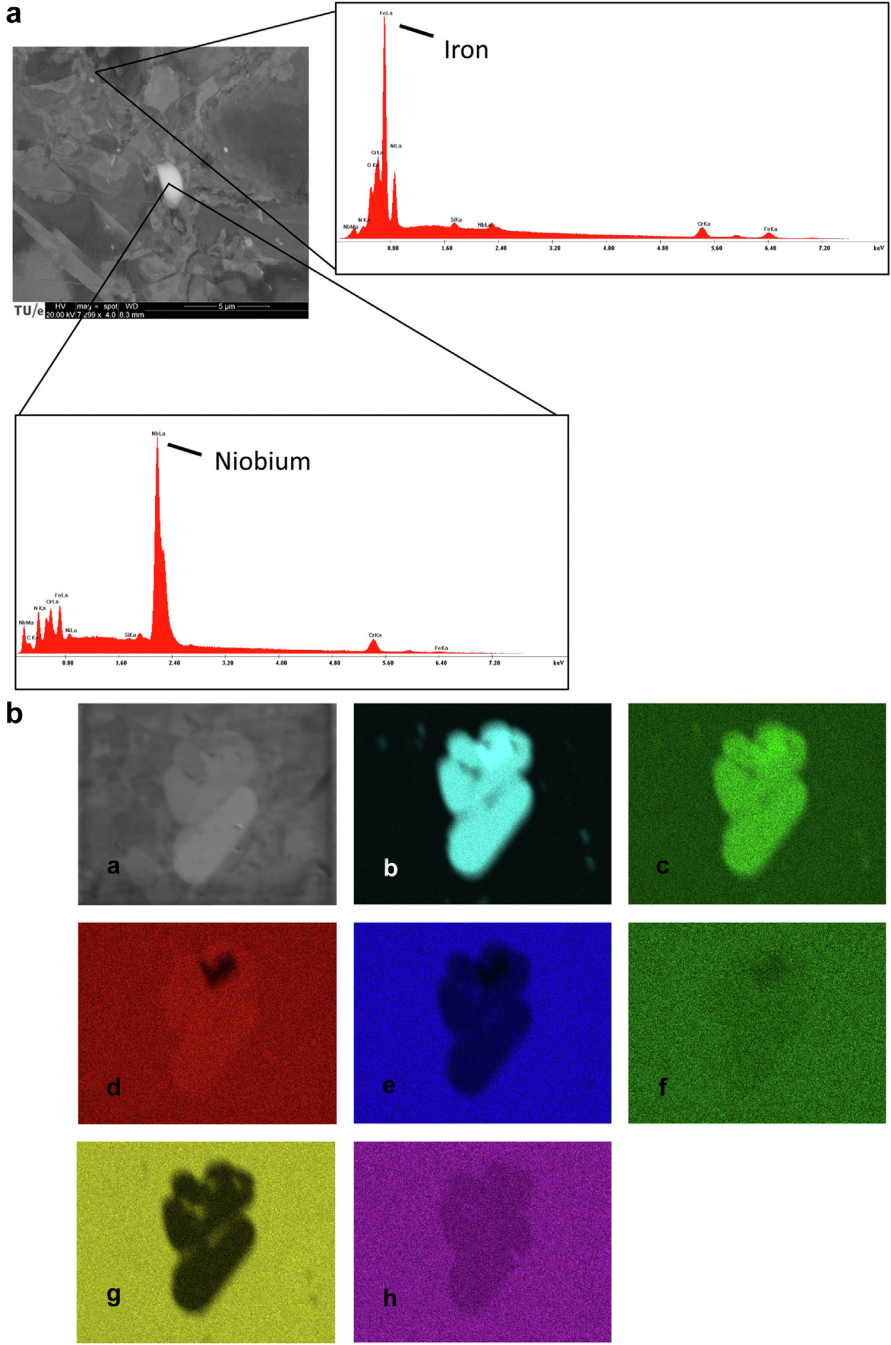
(a) Scanning electron microscopy image with elemental analysis at the indicated niobium-rich location (white particle) and at a control location outside the precipitate. The Nb-rich regions also reveal a higher concentration of nitrogen and, to some extent, chromium. (b) Element maps showing the concentration of elements in the Nb-rich phase (most likely CrNbN zeta phase) within the matrix (austenite). A, scanning electron microscopy overview; B, niobium; C, nitrogen; D, chromium; E, iron; F, manganese; G, nickel; H, silicon.

Cross-section B revealed white spots under the scanning electron microscope which appeared to be niobium (Nb)-rich phases on higher magnification (Fig. 6). Energy dispersive spectroscopy was used to identify the different elements with their compositional fraction of the metal alloy and were compared to the ISO standard. At cross-section A, most of the element fractions were in line with the expectations, except for carbon (C) and nitrogen (N), which clearly surpassed the ISO5832-9 specification (Table 2). The scanning electron micrographs and element mapping were also performed at Nb-rich locations (Fig. 7), revealing a higher concentration of N and, to some extent, also chromium (Cr). The C content in these regions is not given, as it is not accurate enough.

**Table 2:** Element analysis using energy dispersive spectroscopy at the stem away from the fractured surface compared (location A) to the ISO5832-9 standard.

Element	C	N	Si	Cr	Mn	Fe	Cu	Ni	Nb	Mo	P	S
ISO5832-9	≤0.06	0.4	≤0.6	20.5	4.0	balance	≤0.20	9.5	0.3	2.4	≤0.025	≤0.003
EDS	**0.36**	**0.67**	0.49	20.80	3.73	62.01	0.18	9.28	0.17	2.14	0.09	0.09

C, carbon; Cr, chromium; Cu, copper; EDS, energy dispersive spectroscopy; Fe, iron; Mn, manganese; Mo, molybdenum; N, nitrogen; Nb, niobium; Ni, nickel; P, phosphorus; S, sulfur; Si, silicon.

The deviating values are shown in bold.

**Figure 8:**
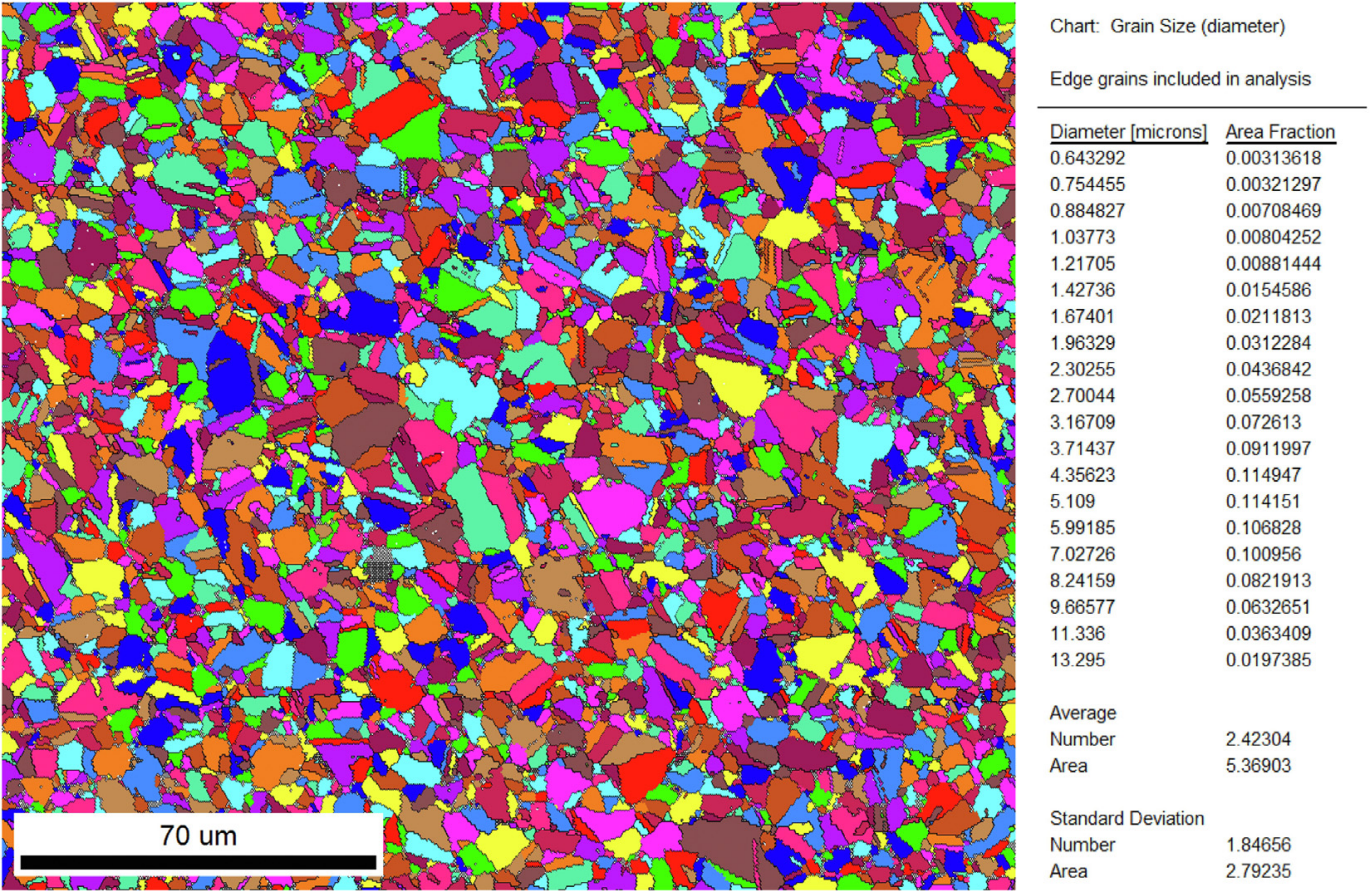
Grain size analysis using orientation imaging microscopy (colors only indicate the existence of the different grains).

Orientation imaging microscopy was used at cross-section B to analyze the grain structure and orientation, allowing to visualize the microstructure of the base material (austenite). The average grain size was ~5 microns. The global microstructure was visualized using electron backscatter diffraction imaging (Fig. 8), whereby each grain is indicated with a separate color to distinguish it from its neighbors. The electron backscatter diffraction also revealed CrNbN Z-phases.

### Fractography

**Figure 9:**
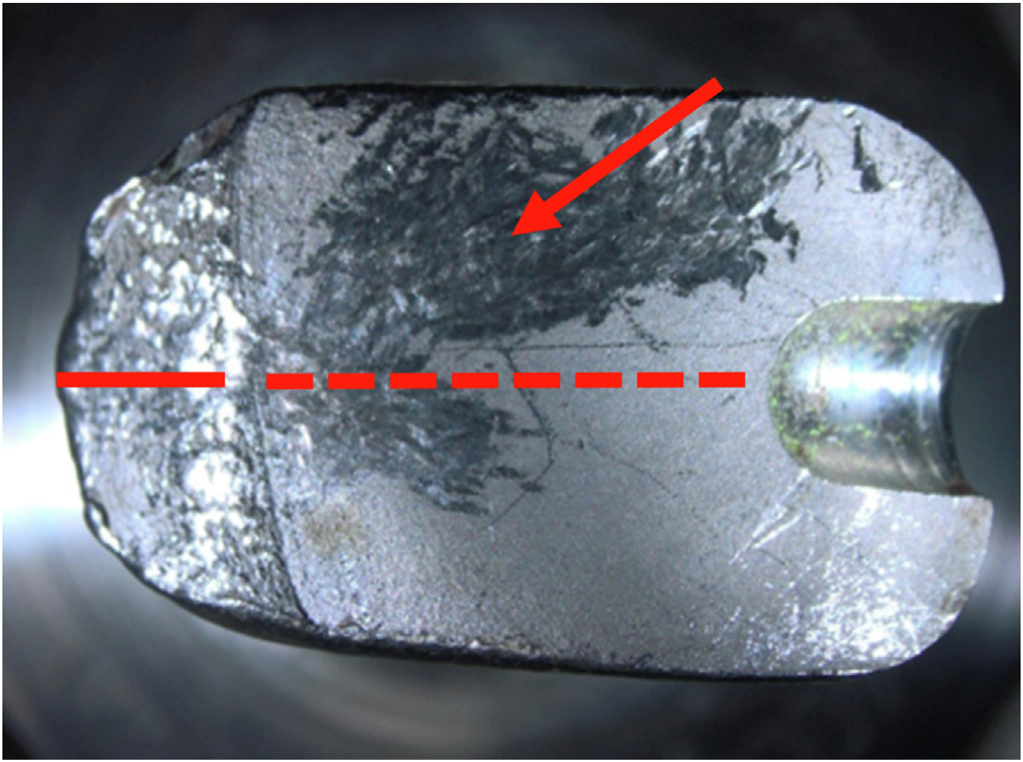
View of the fracture plane. On the right is one half of the introducer stud hole. The dotted line entails the smooth fracture fraction with beach marks. The uninterrupted line is where the stem ultimately fractured. The arrow points to the abrasion marks due to the stem parts moving on to each other in vivo.

The fracture plane and the microstructure of the material in the fracture plane were initially assessed using stereomicroscopy. The fracture plane mainly consisted of a smooth surface with striations and beach marks, which are characteristic for fatigue failure. The residual crack surface was rough and dimpled, which is the surface where the prosthesis ultimately failed under loading. As the 2 stem parts were in frictional contact with each other, surface damage in the form of abrasions emerged (Fig. 9).

**Figure 10:**
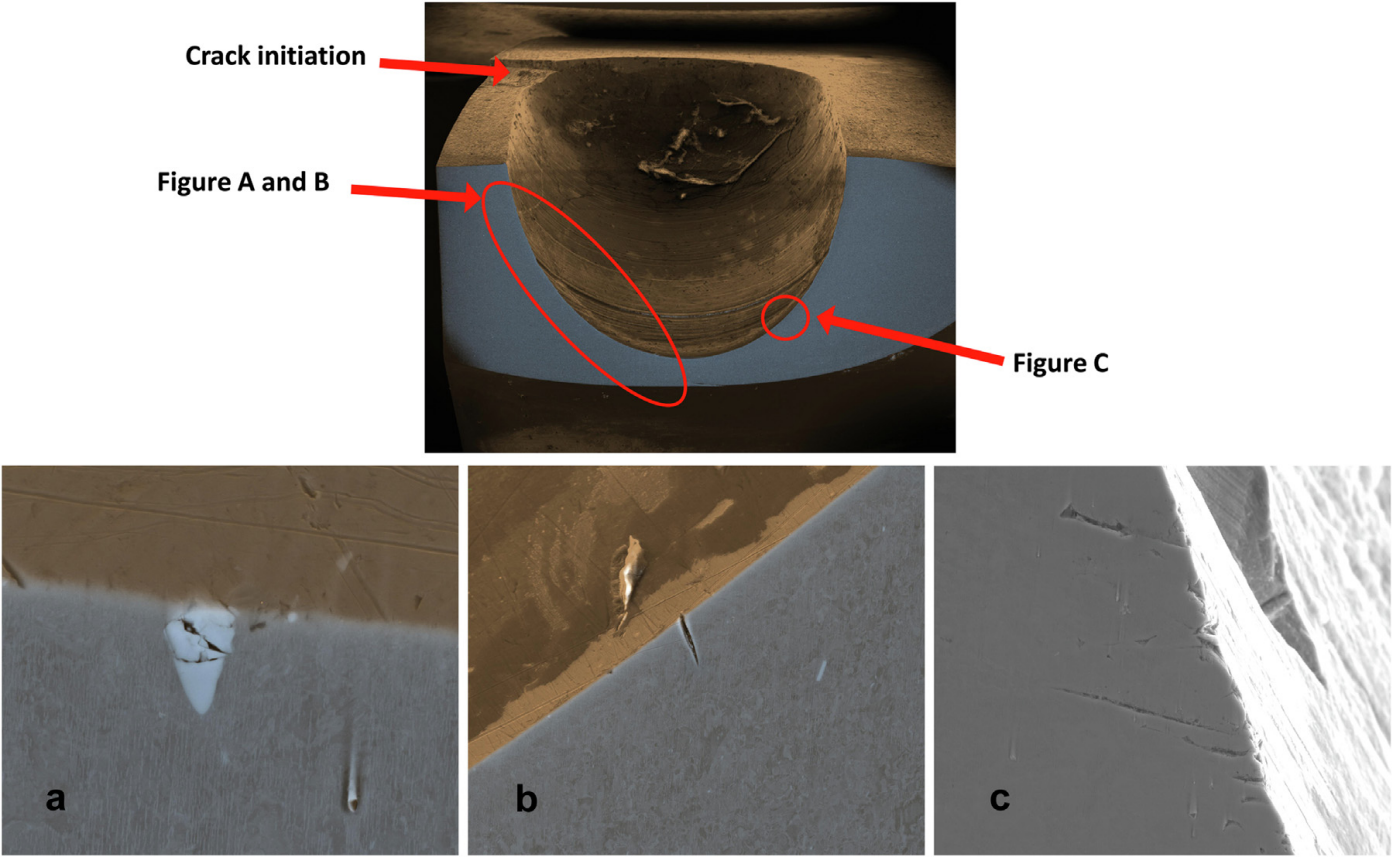
Fractography of the introducer stud hole. Colored scanning electron microscopy imaging combining secondary electron and backscattered electron imaging. (a) A clear example of a CrNbN phase — precipitate — at the edge of the stud hole with microcracks inside; (b) and (c) microcracks.

The introducer stud hole was further analyzed using secondary electron and backscattered electron analyses. The presence of multiple smaller cracks near the stud hole and near or at the Nb-rich precipitates was identified (Fig. 10).

## Discussion

This case report is not the first to describe a fracture that initiated at the introducer hole of an Exeter stem [2,3,7,8,[13], [14], [15], [16], [17]]. Nor is it the first to use metallography [2,7,15,16], fractography [7,15,16], and/or radiography [2,3,7,8,[13], [14], [15], [16], [17]] to analyze such a failure case. However, it is the first report to use these modalities together with histopathology and biochemistry to analyze and synthesize this very rare complication of THA.

### Patient factors

The patient in this case report is not a typical senior with low physical demand. Instead, it is a young, tall, heavy, and active male. Not coincidentally, this seems to be the category at risk for a fracture of the femoral prosthesis itself, particularly at the neck [2].

Perhaps the odds of the combined presence of unfavorable patient and implant determinants in a single patient are generally very low. However, the consequence of implant failure on individual level, such as the risk of impaired clinical outcomes and infection after revision surgery, could give rise to high morbidity and even mortality and is therefore unacceptable [18,19]. Moreover, the demand for THA was calculated to rise with a projected 71% in 2030 [6], and most of the growth is anticipated in patients aged 45-55 years [20]. The projected obesity development in this age group will be alarmingly high [21]. Interestingly, there is no upper weight limit for use of Exeter stems [8,13]. It can, therefore, be hypothesized that the contribution of nontraumatic fractures of the femoral stem will disproportionately grow in the nearby future. Improving the design of the implant with special focus on young, heavy, and active patients could aid in reducing the projected increase of revision surgeries. Furthermore, careful consideration should be given to the risk of implant fracture when decisions are being made about primary or revision hip surgery in morbidly obese patients [17].

### Surgical factors

According to Jazrawi et al., there are 3 surgical factors that can result in failure of a cemented stem [22]:

1) high stress due to an undersized prosthesis,
2) cantilever bending resulting from good distal fixation in the presence of an inadequate proximal mantle, and
3) varus orientation of the stem.

In this case, the prosthesis was neither undersized nor was it placed in varus. Inadequate medial support combined with a well-fixed distal portion of the stem mostly results in a body fracture [2], which makes this factor less relevant with regard to the current case. Even more so, an adequate cement mantle was present during revision surgery.

In addition to the aforementioned surgical factors, Swarts et al. suggest that fretting corrosion or wear can also contribute to the implant failure [7]. We found the fractured trunnion well fixed to the head, no local display of metallosis, and no metal particles present.

It has been suggested that the cause of neck fractures lies in the use of large heads with augmented offsets, in combination with overweight patients [3]. However, this mechanism has been described for causing basal neck fractures instead of an introducer hole fracture [8], although mechanically this would also increase the stresses around the introducer stud hole.

Nonetheless, as more Exeter stems are placed in obese patients with a variety of offset, stem, and head sizes, surgeons must be aware of the risk of stem fracture. Wherever possible, the largest stem that will fit the femur with correct size offset should be used, and heads with larger necks should be avoided [8].

In present case, we performed a cement-in-cement revision, which implied the implantation of an undersized stem. Indeed, in light of the acquired knowledge outlined previously and in previous reports [14], one could question our choice for the cement-in-cement revision with regard to the increased fracture risk (of the undersized stem). However, a complete revision would mean chiseling out the entire intact cement mantle to allow for the implantation of an appropriately sized uncemented stem. Therefore, we feel that the relatively less invasive surgical procedure combined with the proven cement-in-cement concept of the Exeter stem [23] supplies sufficient substantiation for the choice made at the time.

### Implant factors

Overall, the material reveals a rather homogeneous microstructure. The heterogeneities that stand out are the Nb-rich phases. Niobium is typically used in austenitic stainless steels as a stabilizing element. The higher C content entails a risk for the formation of Cr carbides, which tend to deplete the austenitic matrix from C along the grain boundaries, possibly resulting in corrosion (pitting corrosion, particularly when subjected to chloride ions). The Nb has a stabilizing role by preventing the formation of Cr carbides. The addition of Nb to stainless steels is known to induce the precipitation of Nb carbonitrides, in different sizes and shapes [24]. The energy dispersive spectroscopy results indicate that the grain of the phase examined here was indeed Nb(C,N) precipitates. These precipitates have a high hardness.

From the experimental analysis, there are indications that the interfaces of the precipitates are rather weakly bonded. This also means that the precipitates are easily “broken out,” for example, during mechanical processing treatments. Voids were also observed at and near the precipitates.

The fractography revealed the presence of multiple smaller cracks near the stud hole and near or at the Nb-rich precipitates. The present study indicates that the manufacturing of the stud hole may well have induced these cracks, or at least initiated them. As the stem was removed by means of a separately drilled hole in the stem, the introducer hole remained untouched. We therefore postulate that cracks in the introducer stud hole were formed during the manufacturing of the prosthesis. Most likely, the Nb-rich precipitates (CrNbN zeta phase) are abrasively removed upon machining the stud hole, leaving behind regions of stress concentrations and tiny microcracks. These likely serve as initiators for fatigue cracking.

Beach marks and fatigue striations clearly indicate a fatigue failure mode [15]. A hole or crack in the surface of a metal results in a substantial increase of the stress level in the surrounding material. Hence, the combined presence of this microscopic fissure and the introducer hole is expected to be the prime cause of the fatigue “crack” initiation for this failure case [25,26].

Moreover, the Exeter stem design consists of a lever-arm geometry. From a pure mechanical perspective, it is, therefore, remarkable that the relatively large introducer hole is manufactured at its current location where, provided that the stem is adequately cemented in the femur shaft, the bending moment is large. From a mechanical perspective, it would be relatively easy to propose a solution that does not entail such undesirable stress concentrations. For instance, future stem designs could reduce the normal stress due to the bending moment at the introducer hole by placing it more lateral, that is, at the wider part of the stem, such as with the small 30-mm cemented Exeter [27]. Another solution could be to reverse the engaging principle between the introducer and the hole: a protruding mounting piece on the stem instead of a hole. Although implant design changes would mandate costly and laborious amendments to regulatory bodies, they could prevent serious adverse events.

Blood metal ion concentration levels were elevated when the patient presented with the failed prosthesis. However, the observed levels were all lower than the highest values found in well-functioning Exeter prostheses after 1 year of follow-up [12]. Moreover, they were lower than the threshold for which additional research is indicated [10] and lower than values found in well-functioning metal-on-metal protheses which typically produce more wear particles [11]. Therefore, blood metal ion concentration levels are not to be used for monitoring or predicting impending implant failures nor can they define the metal fatigue process.

The histologic patterns of joint endoprosthesis particle disease would consist of the accumulation of macrophages and multinuclear giant cells in the neosynovium. Although abrasion particles differ in quality, quantity, and size subject to the materials and the intensity of the mechanical stress involved, most wear particles are not diagnosed by light microscopy examination becaue of their small size [28]. As the tissue sample of the capsule showed only some eosinophilic granulocytes mixed with some nuclear debris, a few giant cells, and no metal particles, it is likely that the fracture process was slowly progressive.

## Summary

Failure of the metal alloy of a hip prosthesis is an extremely rare but serious adverse event. In this case, the combination of high mechanical stress and an alloy artifact at a mechanically vulnerable location provided the “perfect storm” which ultimately led to the cascade of crack initiation, metal fatigue, and implant fracture. Although rare, this was not the first case and, most certainly, will not be the last. Simple design adaptations could and should prevent the current fracture from happening in the future, especially since younger and heavier patients will demand hip arthroplasty in the future.

## Conflicts of interest

The authors declare that they have no known competing financial interests or personal relationships that could have appeared to influence the work reported in this article.

## Informed patient consent

The author(s) confirm that informed consent has been obtained from the involved patient(s) or if appropriate from the parent, guardian, power of attorney of the involved patient(s); and, they have given approval for this information to be published in this case report (series).

## Appendix A Supplementary data

Conflict of Interest Statement for Fransz

Conflict of Interest Statement for Geers

Conflict of Interest Statement for Jeuken

Conflict of Interest Statement for van Maris

Conflict of Interest Statement for ten Broeke

## References

[1] Learmonth I.D., Young C., Rorabeck C. (2007). The operation of the century: total hip replacement. Lancet.

[2] Bolland B.J.R.F., Wilson M.J., Howell J.R., Hubble M.J.W., Timperley A.J., Gie G.A. (2017). An analysis of reported cases of fracture of the universal Exeter femoral stem prosthesis. J Arthroplasty.

[3] Reito A., Eskelinen A., Pajamäki J., Puolakka T. (2016). Neck fracture of the Exeter stem in 3 patients: a cause for concern?. Acta Orthop.

[4] Bozic K.J., Kurtz S.M., Lau E., Ong K., Vail T.P., Berry D.J. (2009). The epidemiology of revision total hip arthroplasty in the United States. J Bone Joint Surg Am.

[5] Ulrich S.D., Seyler T.M., Bennett D. (2008). Total hip arthroplasties: what are the reasons for revision?. Int Orthop.

[6] Sloan M., Premkumar A., Sheth N.P. (2018). Projected volume of primary total joint arthroplasty in the U.S., 2014 to 2030. J Bone Joint Surg Am.

[7] Swarts E., Kop A., Jones N., Keogh C., Miller S., Yates P. (2008). Microstructural features in fractured high nitrogen stainless steel hip prostheses: a retrieval study of polished, tapered femoral stems. J Biomed Mater Res A.

[8] Garala K., Laios T., Lawrence T. (2018). A report of 3 cases of Exeter V40 stem fracture and explanation of possible causes. Hip Int.

[9] Hailer N.P., Garellick G., Karrholm J. (2010). Uncemented and cemented primary total hip arthroplasty in the Swedish Hip Arthroplasty Register. Acta Orthop.

[10] (2017). All metal-on-metal (MoM) hip replacements: updated advice for follow-up of patients. Med Healthc Prod Regul Agency (Mhra).

[11] Sampson B., Hart A. (2012). Clinical usefulness of blood metal measurements to assess the failure of metal-on-metal hip implants. Ann Clin Biochem.

[12] Leyssens L., Vinck B., Van Der Straeten C., Wuyts F., Maes L. (2017). Cobalt toxicity in humans-A review of the potential sources and systemic health effects. Toxicology.

[13] Akinola B., Mahmud T., DeRoeck N. (2009). Fracture of an Exeter stem - a case report. Internet J Orthop Surg.

[14] O'Neill G.K., Maheshwari R., Willis C., Meek D., Patil S. (2011). Fracture of an Exter 'cement in cement' revision stem: a case report. Hip Int.

[15] Facek M., Khatib Y., Swarts E. (2016). Prosthetic fracture of a cemented Exeter femoral stem. Reconstr Rev.

[16] Moloney D.P., Hurley R.J., Harty J., Guerin S. (2019). History, treatment and analysis of a rare form of Exeter stem fracture. BMJ Case Rep.

[17] Hamlin K., MacEachern C.F. (2014). Fracture of an Exeter stem: a case report. JBJS Case Connect.

[18] Weber M., Renkawitz T., Voellner F. (2018). Revision surgery in total joint replacement is cost-intensive. Biomed Res Int.

[19] Berstock J.R., Beswick A.D., Lenguerrand E., Whitehouse M.R., Blom A.W. (2014). Mortality after total hip replacement surgery: a systematic review. Bone Joint Res.

[20] Kurtz S.M., Lau E., Ong K., Zhao K., Kelly M., Bozic K.J. (2009). Future young patient demand for primary and revision joint replacement: national projections from 2010 to 2030. Clin Orthop Relat Res.

[21] Sturm R., Hattori A. (2013). Morbid obesity rates continue to rise rapidly in the United States. Int J Obes (Lond).

[22] Jazrawi L.M., Della Valle C.J., Kummer F.J., Adler E.M., Di Cesare P.E. (1999). Catastrophic failure of a cemented, collarless, polished, tapered cobalt-chromium femoral stem used with impaction bone-grafting: a report of two cases. J Bone Joint Surg Am.

[23] Woodbridge A.B., Hubble M.J., Whitehouse S.L., Wilson M.J., Howell J.R., Timperley A.J. (2019). The exeter short revision stem for cement-in-cement femoral revision: a five to twelve year review. J Arthroplasty.

[24] Ha V.T., Jung W.S. (2011). Niobium carbo-nitride precipitation behavior in a high nitrogen 15Cr-15Ni heat resistant austenitic stainless steel. Met Mater Int.

[25] Lee E.W., Kim H.T. (2001). Early fatigue failures of cemented, forged, cobalt-chromium femoral stems at the neck-shoulder junction. J Arthroplasty.

[26] Woolson S.T., Milbauer J.P., Bobyn J.D., Yue S., Maloney W.J. (1997). Fatigue fracture of a forged cobalt-chromium-molybdenum femoral component inserted with cement. J Bone Joint Surg Am.

[27] Stryker. Exeter® V40® femoral stem surgical technique. https://www.strykermeded.com/media/1216/exeter-using-modular-broach-surgical-technique.pdf. [accessed 27.08.21].

[28] Krenn V., Morawietz L., Perino G. (2014). Revised histopathological consensus classification of joint implant related pathology. Pathol Res Pract.

